# Optical soliton molecular complexes in a passively mode-locked fibre laser

**DOI:** 10.1038/s41467-019-08755-4

**Published:** 2019-02-19

**Authors:** Z. Q. Wang, K. Nithyanandan, A. Coillet, P. Tchofo-Dinda, Ph. Grelu

**Affiliations:** 1 Laboratoire Interdisciplinaire Carnot de Bourgogne, UMR 6303 CNRS, Université Bourgogne Franche-Comté, 9 Av. A. Savary, F-21078 Dijon, France; 2 College of Electronic and Optical Engineering and College of Microelectronics, Nanjing University of Posts and Telecommunications, 210023 Nanjing, China; 3 LIPhy–Laboratoire Interdisciplinaire de Physique, UMR 5588 CNRS, Université Grenoble Alpes, F-38400 Saint Martin d’Hères, France

## Abstract

Ultrashort optical pulses propagating in a dissipative nonlinear system can interact and bind stably, forming optical soliton molecules. Soliton molecules in ultrafast lasers are under intense research focus and present striking analogies with their matter molecules counterparts. The recent development of real-time spectral measurements allows probing the internal dynamics of an optical soliton molecule, mapping the dynamics of the pulses’ relative separations and phases that constitute the relevant internal degrees of freedom of the molecule. The soliton-pair molecule, which consists of two strongly bound optical solitons, has been the most studied multi-soliton structure. We here demonstrate that two soliton-pair molecules can bind subsequently to form a stable molecular complex and highlight the important differences between the intra-molecular and inter-molecular bonds. The dynamics of the experimentally observed soliton molecular complexes are discussed with the help of fitting models and numerical simulations, showing the universality of these multi-soliton optical patterns.

## Abstract (web summary)

It has recently been shown that optical solitons can form stably bound states, so-called soliton molecules. Here, Wang et al. demonstrate stable soliton molecule complexes and explore the different bonds represented by the inter- and intra-molecular coupling.

## Introduction

The soliton can be considered as a central concept promoting analogies between matter and light as it combines, in a striking manner, wave and particle-like behaviours. As a matter of fact, after being discovered in the context of hydrodynamics, solitons were found in plasma physics and optics, as well as in most areas of nonlinear science, including DNA mechanical waves and Bose-Einstein condensation^1^. A soliton is a wave packet localised in time and/or space that results from a balance between linear and nonlinear physical effects. As such, the soliton possesses an inherent stability that allows it to propagate without spread and distortions over large distances, in contrast to linear wave packets that are subjected to dispersion. In addition, the soliton maintains the integrity of its waveform in the event of collisions and noise perturbations. Therefore, optical temporal solitons have long been considered as valuable assets for the upgrade of long-haul optical communications^2^ and are currently driving accelerated research in the context of ultrashort pulse generation in laser cavities, where the concept has been extended to encompass the solitary waves of dissipative systems, namely “dissipative solitons”^3^. With dissipative solitons, remarkable new properties, which were mostly absent in integrable systems, have been highlighted. Those properties include the ability to form robust multi-soliton bound states, currently termed “soliton molecules”^3–10^. An optical cavity constitutes an ideal propagation medium to study multiple soliton interactions, since even ultraweak interactions can be revealed through the virtually unlimited propagation time^11–15^. Once formed, stable optical soliton molecules will propagate almost indefinitely around a mode-locked laser cavity^6,7^. In other scenarios, they can evolve under mutual collisions, resulting in possible dissociations or in the synthesis of new molecules^16,17^, form various “isomers”^18^, and even “polymerise” into macromolecules and soliton crystals^19^, comprising up to thousands of bound soliton pulses^20^.

These striking properties fuel the analogy with matter molecules, though matter and optical soliton molecules remain fundamentally different physical entities. Optical soliton molecules are based on the existence of attractors for the underlying nonlinear dynamical system. As dissipative patterns, they maintain themselves as long as the external pump source is present^3^. Nevertheless, more interesting parallels between light and matter molecules can be drawn. For instance, vibrating states of soliton molecules in ultrafast lasers were anticipated from 2006, but their experimental confirmation had been first mitigated by the lack of real-time accurate ultrafast measurements^21–26^. Possible doubts about the existence of vibrating and oscillating soliton molecules ended in 2017, when two independent studies unveiled the real-time evolution of the internal motions within two-soliton and three-soliton molecules, by employing an advanced spectro-temporal measurement called the time-stretch dispersive Fourier-transform (DFT) technique^9,10^. In a soliton molecule, the most relevant degrees of freedom of the internal dynamics are the relative temporal separations among solitons, as well as their relative phases^4,5^. The above studies unveiled a variety of oscillation and vibration dynamics, including phase-and-separation oscillations, phase-dominated oscillations, and sliding phase dynamics. At this point, we stress that albeit the analogy, vibrating optical soliton molecules remain fundamentally different from vibrating matter molecules, as the former do not exhibit the quantisation of the latter. Instead, the onset of vibrating and oscillating soliton molecules typically follows a Hopf-type bifurcation^3^.

Aware of these fundamental differences, the salient question that naturally arises is to guess how far the analogy between optical soliton bound states and matter molecules can go, considering their structures, as well as their dynamics^27^. Overlapping soliton pulses (namely, when the temporal separation between solitons is of the order of a few soliton widths) are likely to form strong bonds, whereas more distant solitons and molecules are expected to form weaker bonds. We were actually guided by this analogy with the interaction between atoms to find and characterise the soliton molecular complexes.

In the present communication, by employing an ultrafast fibre laser setup whose output is spectrally analysed on a real-time shot-to-shot basis, we demonstrate that two basic molecules, each made up of a pair of solitons, can bind stably so as to form what we define here as a “2 + 2 soliton molecular complex” (SMC), i.e., an entity in which the bond between the basic molecules may differ by its nature and its dynamics from the bond between the two solitons of each basic molecule. We show that the experimental dynamics of a 2 + 2 SMC can be interpreted thanks to a simple analytical description and reproduced with the help of numerical simulations. The 2 + 2 SMC thus reveals a new aspect of the universality of optical soliton molecules, because it appears as the optics version of the well-known molecular complexes in condensed-matter physics.

## Results

### Real-time spectral monitoring

The general challenge consists in recording the evolution of an ultrashort pulse waveform over successive cavity roundtrips, which typically means at multi-MHz frame rates for most mode-locked lasers, and access phase and amplitude information within sub-picosecond accuracy. This issue is accentuated for unamplified laser output pulses, with pulse energy in the nanojoule range or below: self-referenced nonlinear methods such as single-shot optical autocorrelation cannot be efficiently implemented. Therefore, there has been remarkable efforts in developing heterodyne techniques, which involve nonlinear wave mixing with an intense frequency-chirped synchronised pump, leading to time-lens systems that recently proved their ability to retrieve phase and amplitude information of generic pulse waveforms^28,29^. Nevertheless, in our situation, considering that all solitons will have the same waveform, carved by a common dissipative soliton attractor^3^, it is considerably simpler and as efficient to rely on DFT, a recently developed linear technique^30^ that allows to implement the proven phase retrieval techniques of spectral interferometry ^31,32^.

The DFT measurement method maps the optical spectrum of the laser output onto a temporal waveform that is directly read out on a real-time oscilloscope. This is achieved by propagating linearly the attenuated laser output pulses through a highly-dispersive medium. Consequently, the pulsed waveform is stretched and, provided that the total accumulated dispersion is large enough for the pulse propagation to satisfy the far-field condition, the stretched waveform will represent the spectral intensity of the initial pulse waveform. Therefore, by carefully designing the dispersive link, the laser pulses at all successive cavity roundtrips can be spectrally analysed in real-time at multi-MHz rates, in contrast to the slow, averaged spectral information generally provided by optical spectrometers^30,33–36^. Though a convenient and fast-spreading real-time spectral measurement method, DFT conceals a few nontrivial issues related to the spectro-temporal mixing of its carried optical information. Such issues are particularly salient, for instance, when analysing the self-starting short pulse dynamics^34^, especially when it involves multiple pulses^36,37^. Beyond the simple notion of spectral resolution, the application of the techniques of spectral interferometry are also limited by the current recording features of fast electronics, as well as by the number and the distribution of soliton pulses. The number of pulses that can be followed in real time, under given conditions and pulse distributions, is still a challenging question, which we have addressed here by extending the characterisation to soliton molecular complexes.

**Fig. 1:**
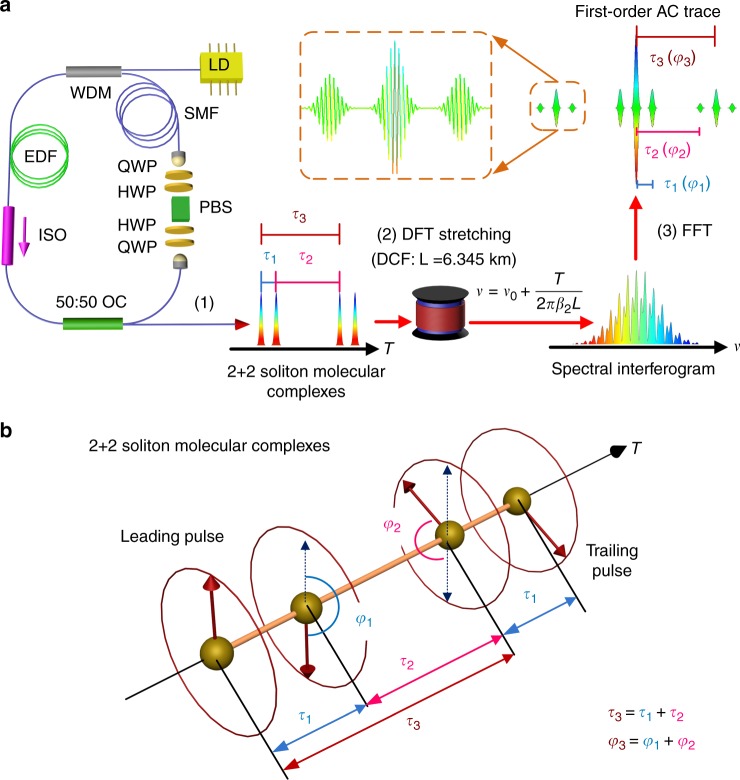
Schematic of the generation and real-time characterisation of a soliton molecular complex. **a** A complex of two ultrafast soliton-pair molecules emitted from the fibre ring laser (1) is characterised by the relative temporal separations *τ*_*i*=1,2,3_ and phases *φ*_*i*=1,2,3_. To measure these internal degrees of freedom in real time by use of the dispersive Fourier-transform (DFT) method, the molecular complex is attenuated so as to propagate linearly (2) through a long length *L* of highly-dispersive optical fibre (DCF, dispersion ***β***_2_). The temporal overlap between the pulses dispersed in the far-field regime results in an interferogram, through the relationship between local time *T* and frequency ***v***. The interferogram contains the information about the relative phases and temporal separations, which is revealed by fast Fourier-transforming (FFT) successive interferograms (3) on a shot-to-shot basis, yielding first-order autocorrelation (AC) traces. As a result, we access the ultrafast evolution of the soliton molecular complex with a frame rate of tens of MHz–the cavity free spectral range. EDF erbium-doped fibre, WDM wavelength-division multiplexer, LD laser diode, SMF single mode fibre, PBS polarising beam splitter, ISO optical isolator, OC optical coupler, QWP quarter-wave plate, HWP half-wave plate. See Methods for laser cavity description. **b** Graphical representation of the internal degrees of freedom of a soliton molecular complex. The solitons are represented by phasors, distributed along a temporal axis according to their relative temporal separations *τ*_i_. The leading soliton is a fixed phasor, pointing upward in the diagram, which serves as a reference for the succeeding phasors. This allows a clear representation of the internal dynamics of the soliton molecular complex

When instead of a single pulse, the laser generates a soliton molecule comprising two solitons—namely, a soliton pair—separated by a time *τ* and having a relative phase *φ*, the two internal degrees of freedom (*τ*, *φ*) of the molecule can be easily retrieved from an optical spectral recording: the spectral fringe period *Δν* reflects the pulse temporal separation *τ*, through *Δν* = 1/*τ*, while the spectral offset of the fringes with respect to the spectral envelope, *δν*, yields the relative phase as: *φ* = 2*π δν*/Δ*ν*^38^. For a soliton molecule containing more than two pulses, the information concerning the relative phases and temporal separations between constituent solitons can be retrieved through the methods of spectral interferometry, under certain conditions^31,32^. The retrieval of internal phase and separation dynamics in the case of three-soliton optical molecules was recently undertaken^10^. Here, we generate and characterise several (2 + 2) SMCs, as illustrated in Fig. 1a. The phase and temporal separation information can be extracted through the Fourier-transform of each interferogram (see also Methods). By analysing this way successive spectral interferograms, we can resolve the ultrafast dynamics of soliton interactions in real-time. Concerning soliton molecules in general, we point out that a common dynamical attractor of the nonlinear dissipative system—the ultrafast laser—will carve each pulse profile in a similar way, so that the most relevant internal variables are the relative phases and temporal separations among solitons. This holds as long as the pulses do not strongly overlap. Therefore, we propose with Fig. 1b a phasor representation of the solitons, in order to visualise more easily the internal dynamics of the molecular complex, as it will be shown in the following. Note that this representation does not picture polarisation, as the latter degree of freedom is frozen by virtue of the intracavity polarizer, noted as PBS in Fig. 1a.

### Experiments

The experimental setup for generating bound soliton molecules is an erbium-doped passively mode-locked fibre laser, sketched on Fig. 1a, which is optically pumped by a 980-nm laser diode and emits at a wavelength ~1.55 μm (details in the Methods section). Since the laser is operated in the anomalous dispersion regime, soliton pulse shaping will limit the single-pulse energy to less than 100 pJ, leading to the generation of multiple pulses for pump powers greater than 100 mW, typically. Mode locking relies on the nonlinear polarisation evolution technique, which allows tuning the nonlinear transfer function by simply rotating intra-cavity phase-retarding plates, thus modifying the interactions among pulses ^39,40^.

Mode-locked lasers generally present an important hysteresis with respect to the pumping power, which is exacerbated in the case of multiple-pulse dynamics, where it leads to multi-stability^41,42^. We use these physical phenomena in an experimental procedure which allows us to generate several types of SMCs in a reproducible way. We start at a high pumping power of 400–450 mW, where self-starting mode locking is accompanied with the generation of 6–8 pulses per cavity roundtrip. Subsequently, we annihilate pulses one by one by decreasing the pump power, to retain four pulses. In the following case, these four pulses are obtained at a pump of 371 mW. In the meantime, we also tune the pulse interactions, through a small rotation on the polarisation waveplates. Pulse self-assembly takes place during this process, forming two soliton pairs. By further fine tuning the waveplates orientations and reducing the pump to 317 mW, we prepare a robust (2 + 2)—SMC consisting of two bound soliton-pairs.

**Fig. 2:**
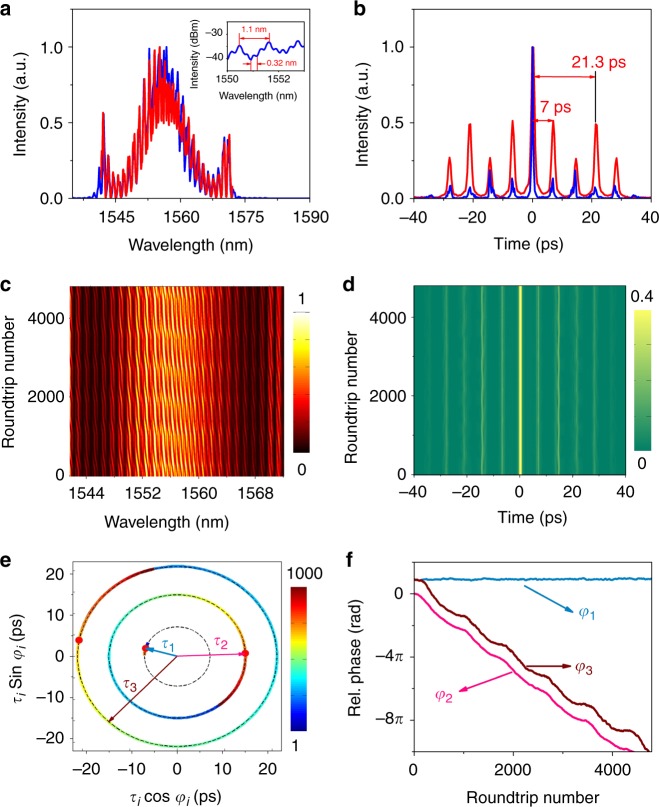
Characterisation of a 2 + 2 soliton molecular complex following sliding-phase dynamics. **a** Optical spectrum, as directly recorded by the multi-shot optical spectrum analyser (blue curves), and as the average of 4800 consecutive single-shot spectra (in red). Inset: Zoom-in showing the spectral periods. **b** Second-order multi-shot-averaged autocorrelation trace (red curve), along with one example of single-shot first-order autocorrelation trace (blue curve) obtained by Fourier-transform of a single-shot spectrum. **c** 2-D contour plots of 4800 consecutive single-shot spectra (spectral intensity in colour scale). **d** Evolution of the intensity of the Fourier-transforms of single-shot spectra, namely first-order single-shot autocorrelation traces (intensity in colour scale). **e** Trajectories of the internal degrees of freedom in the interaction plane, namely the inter-pulse separations *τ*_*i*=1,2,3_ and relative phases *φ*_*i*=1,2,3_. The roundtrip number is displayed in colour scale. **f** Evolution of relative phases between solitons *φ*_*i*=1,2,3_ as a function of the roundtrip number

The averaged optical spectrum and autocorrelation traces shown in Fig. 2a, b reveal the existence of two matching spectral and temporal intervals, corresponding to a 7-ps pulse separation within each soliton-pair molecule and a 21.3-ps separation between the two soliton-pair molecules. This SMC propagates indefinitely round the laser cavity. Therefore, to address the question of whether the two characteristic times are associated with two different bonds, we employ real-time spectral monitoring to characterise each bond type through its specific nonlinear dynamics. The real-time spectral interferogram, recorded for 4800 successive cavity roundtrips, is shown in Fig. 2c. Whereas it displays the two different systems of spectral fringes as expected, we see that the long-period fringe system (period 1.1 nm) is stationary, whereas the short-period one is sliding toward higher frequencies as roundtrips are increased. This is an indication of the major difference between the two bonds, which follow different dynamics. The calibration and accuracy of the DFT spectral measurements are checked by comparing, on Fig. 2a, the average of 4000 consecutive spectra with the averaged spectrum recorded by the optical spectrum analyser (OSA).

To get a quantitative dynamical picture, we retrieve the relative phases and temporal separations within the SMC. For that purpose, the Fourier-transform of each DFT single-shot spectrum, which is equivalent to a first-order optical autocorrelation (AC), is computed and yields the relative temporal and phase separations among the soliton molecule. One example of such AC trace is provided with Fig. 2b and the evolution of these first-order autocorrelation traces is shown in Fig. 2d. Hereafter, we use the term ‘separation coordinates' to refer to a set of variables (*τ*, *φ*), where *τ* and *φ,* respectively, represent the separations between the temporal positions and the phases of two given solitons of the 2 + 2 SMC. We examine the following three coordinates: (*τ*_1_*, φ*_1_) describes the separation between the two solitons of each soliton-pair molecule. The 2 + 2 SMC considered in the present work are made up of two identical soliton-pair molecules. (*τ*_2_, *φ*_2_) describes the separation between the trailing soliton of the leading soliton-pair molecule and the leading soliton of the trailing soliton-pair molecule, see Fig. 1b. (*τ*_3_, *φ*_3_) designates the separation coordinates between the leading (or the trailing) solitons of the two soliton-pair molecules. We plot the evolution trajectory of the three separation coordinates in the interaction plane of Fig. 2e, and the evolution of the relative phases in Fig. 2f. In Fig. 2e, the red points correspond to the initial values of the retrieved temporal separations and phases. Note that the consistency relationship *τ*_3_ = *τ*_1_ + *τ*_2_ is verified. In the present case, the fixed location of (*τ*_1_, *φ*_1_) indicates that the two solitons composing each soliton-pair molecule are phase-locked with a relative phase close to *π* and keep a fixed temporal separation. The other two locations move in circle, confirming that the relative phases *φ*_2_ and *φ*_3_ are changing while the temporal separation between the two soliton-pair molecules of the 2 + 2 SMC remains fixed. Figure 2f indeed shows that the relative phase *φ*_3_ between soliton-pair molecules is continuously decreasing, with a nonlinear modulation period. The relationship *φ*_3_ = *φ*_1_ + *φ*_2_ is verified, which validates the consistency of our phase retrieval. By extension of the sliding-phase dynamics terminology suggested for soliton-pair molecules^9,24^, the proposed terminology for the currently reported dynamics is “sliding-internal-phase SMC”. A graphical illustration of this dynamics can be seen in the Supplementary Movie 1.

**Fig. 3:**
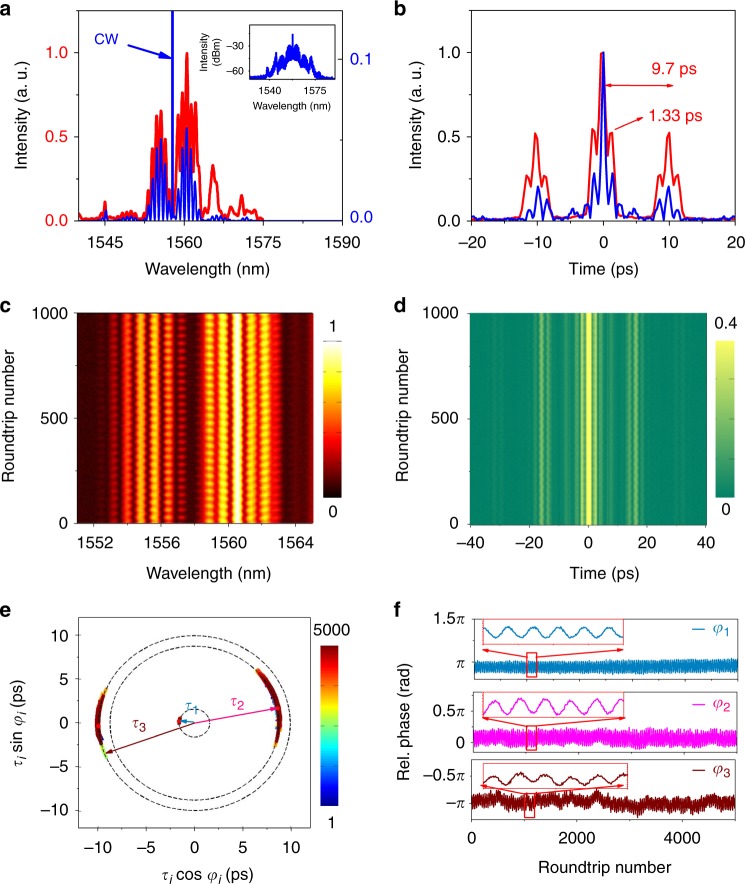
Characterisation of a 2 + 2 soliton molecular complex following oscillating-phase dynamics. **a** Optical spectrum directly recorded by the multi-shot optical spectrum analyser (blue curves) and the average of 1000 consecutive single-shot spectra (in red). CW indicates a residual quasi-continuous-wave component. **b** Second-order multi-shot autocorrelation trace (red curve) and one example of single-shot first-order autocorrelation trace (blue curve). **c** 2-D contour plots of 1000 consecutive single-shot spectra (intensity in colour scale). **d** Evolution of the first-order single-shot autocorrelation trace over 1000 roundtrips (intensity in colour scale). **e** Trajectories of the internal degrees of freedom in the interaction plane: inter-pulse separations *τ*_*i*=1,2,3_ and relative phases *φ*_*i*=1,2,3_. Roundtrip number in colour scale. **f** Evolution of relative phases between solitons *φ*_*i*=1,2,3_ as a function of the roundtrip number. The magnification from 1000 to 1200 roundtrips shows nearly out-of-phase oscillations for *φ*_1_ and *φ*_3_

We now turn to another type of 2 + 2 SMC. In general, lowering solely the pumping power tends to transform a strong bond—corresponding to a stable focus attractor—into a weaker bond, an attractor of limit cycle type^9^. We use the hysteresis of the laser with respect to the pump power to generate two soliton-pair molecules, which form a SMC at a lower pumping power of 240 mW. The averaged optical spectrum and autocorrelation trace are shown in Fig. 3a, b. Compared to the previous case (Fig. 2), the two solitons of each soliton-pair molecule are bound at a shorter separation of 1.33 ps, whereas the temporal separation between two soliton-pair molecules is 9.7 ps. With such a temporal scale difference of nearly one order of magnitude, we are again in the presence of an optical SMC. The average optical spectrum shown in the inset of Fig. 3a exhibits a symmetric interference structure with a central dip, indicating out-of-phase bound solitons within each soliton pair ^4,6^.

In this second SMC case, we observe a rapid evolution of the real-time interferogram, as shown in Fig. 3c. As for the first molecular complex depicted in Fig. 2, the analysis demonstrates that the pulse separations within the second molecular complex are kept nearly constant during the evolution process, as reflected by the calculated first-order single-shot autocorrelation traces shown in Fig. 3d and displayed in the interaction plane of Fig. 3e. However, the dynamics of the relative phases is markedly different in the present case, as revealed by Fig. 3f: the internal dynamics within the SMC is dominated by the oscillation of the relative phases, with a period of 40 cavity roundtrip times. Remarkably, the relative phase *φ*_*1*_ between the two solitons of each soliton-pair molecule is oscillating out of phase with respect to the oscillation of the relative phase *φ*_3_ between the two soliton-pair molecules that form the complex, see the inset in Fig. 3f. The dynamics of the “oscillating-phase SMC” is illustrated by Supplementary Movie 2. Finally, it is worth noting that, whereas the phase oscillation of *φ*_*1*_ is stationary over 5000 roundtrips, the phase oscillation of *φ*_2_ (*φ*_3_) is accompanied by additional fluctuations, probably due to environmental perturbations. This demonstrates again the fact that the bond between the two soliton-pair molecules that constitute the molecular complex is weaker than the bond between the two solitons that make up each soliton-pair molecule.

### Fitting models

**Fig. 4:**
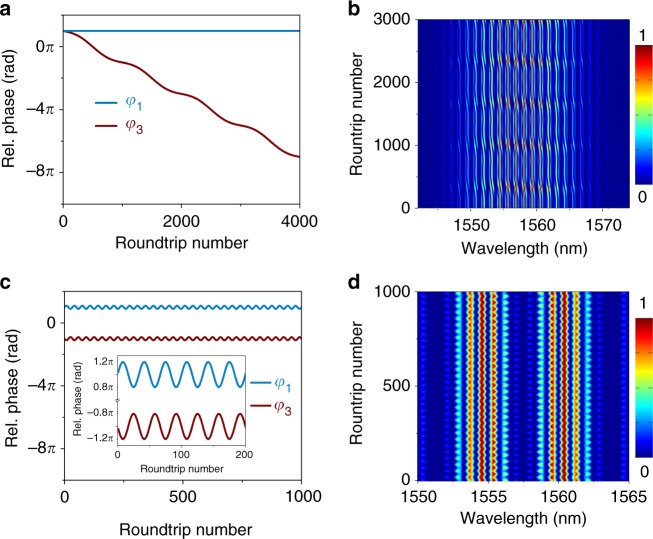
Analytical fits for the dynamics of soliton molecular complexes. **a** Evolution of the relative phases *φ*_1_ and *φ*_3_, defined in Fig. 1b, as a function of the roundtrip number for the sliding phase dynamics and **b** the corresponding evolution of the spectral intensity profile. **c** Phase evolution of *φ*_1_ and *φ*_3_ as a function of the roundtrip number for the oscillating phase dynamics and **d** the corresponding evolution of the spectral intensity profile. The spectral intensity is represented in colour scale in **b** and **d**

In the following, the aim is to reproduce the main observed features of the spectral evolution, by fitting the phase evolution over cavity roundtrips with the help of a simple formula, while keeping the pulse widths and separations fixed. For the first case of a 2 + 2 SMC with a sliding internal phase, we consider a constant phase difference between the two solitons within each soliton-pair molecule (*φ*_1_ = *π*), whereas the phase between the two soliton-pair molecules is modelled by the simple equation *φ*_3_(*z*) = *φ*_0_ *+* *A*_φ_sin(*z*) − *z*. We first fit the phase drift to that observed in Fig. 2f, with *z* = 0.002*πn*, *n* being the roundtrip number, *φ*_0_ = *π*. We then fit the oscillation amplitude which is found to be *A*_φ_ = 0.5*π*. Each soliton of the SMC is chosen to have a Gaussian profile with a temporal width of 300 fs. The choice of the temporal separations, *τ*_1_ = 7 ps and *τ*_3_ = 21.3 ps, correspond to the experimentally retrieved values. Based on these parameters, we model the spectral intensity evolution over 5000 roundtrips in Fig. 4b, with the phase *φ*_*3*_ evolution shown in Fig. 4a. This simple analytical description of the phase evolution reproduces convincingly the results of Fig. 2, confirming the interpretation of the dynamical pulse structure as a sliding-internal-phase SMC. In the second 2 + 2 SMC case, noting the absence of drift and the existence of out-of-phase oscillations, we model the relative phases with the following equations: *φ*_1_(*z*) = *φ*_0_ *+* *A*_φ_sin(*z*), and *φ*_3_(*z*) = *φ*_0 _* − A*_φ_sin(*z*). The phase evolutions are shown in Fig. 4c, where we set the parameters as *φ*_0_ = *π*, *A*_φ_ = 0.1*π* and *z* = 0.06*πn*, with the temporal separations *τ*_1_ = 1.33 ps and *τ*_3_ = 9.7 ps. The evolution of the interferogram over 1000 roundtrips exhibits the same behaviours as in the experiment.

### Numerical simulations

To corroborate the experimental observations, we briefly describe the results of the numerical simulations, obtained using a lumped laser model, where each cavity component is modelled by a separate equation, and the pulse propagates through a concatenated sequence representing the different cavity elements. We use a scalar-field approach as in ref. ^9^, where the saturable absorber is modelled by an instantaneous and monotonous nonlinear transfer function that is characterised by a saturation power *P*_sat_. The gain fibre modelling includes gain saturation, bandwidth limitation and longitudinal dependence of the saturation, quantities depending on the pumping power *P*. More details of the modelling are presented in the Methods section.

**Fig. 5:**
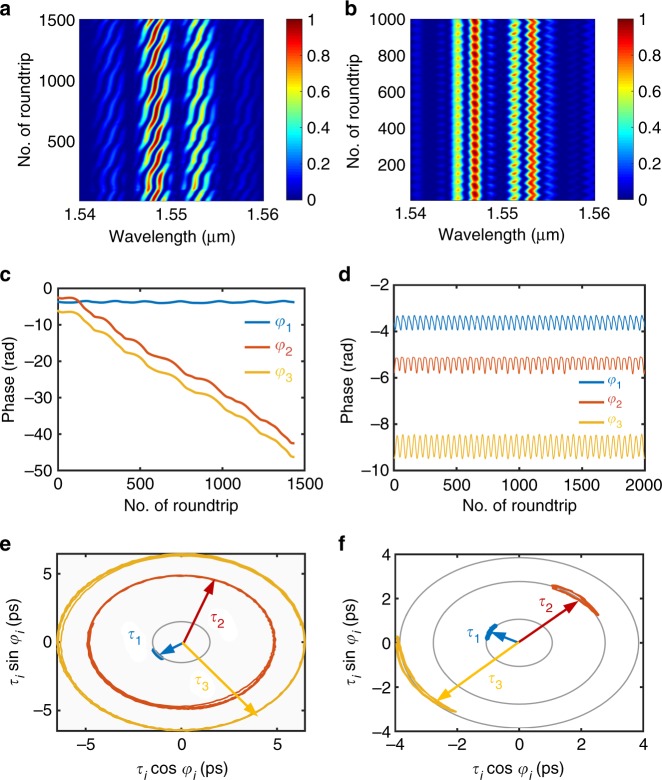
Numerical simulations of the dynamics of soliton molecular complexes. **a**, **c**, and **e** correspond to a soliton molecular complex (SMC) with sliding-phase dynamics, obtained at pump power *P* = 86 mW and saturation power *P*_sat_ = 6 W. **b**, **d**, and **f** feature an oscillating-phase SMC obtained at *P* = 104 mW and *P*_sat_ = 7 W. a and b represent the spectral evolution of the sliding and oscillating SMCs, respectively, over successive cavity roundtrips (Spectral intensity in colour scale). c and d depict the evolution of the internal phases ***φ***_***i***=1,2,3_ within the SMC, in the sliding and oscillating SMC cases, respectively, as a function of the cavity roundtrip time. e and f display the trajectories of the relative temporal separations **τ**_***i***=1,2,3_ and internal phases in the interaction plane representation, for the two different dynamics

For the parameter set defined by the experiments, according to the tuning of *P* and *P*_sat_, we can obtain two soliton-pair molecules that form a molecular complex, characterised by temporal pulse widths in the range of 0.3–0.6 ps and intra-molecular temporal separations in range of 1–3 ps. The inter-molecular separation (between the two soliton-pair molecules) is typically in the range of 3–15 ps. A regime of sliding relative phase within the 2 + 2 SMC, qualitatively close to the experimentally reported dynamics, is obtained for *P* = 86 mW and *P*_sat_ = 6 W as shown in Fig. 5a. In this SMC, the intra-molecular separation is 1.5 ps and the inter-molecular one is 6.5 ps. It is evident from Fig. 5c that the inter-molecular relative phase evolves over time with a given nonlinear modulation period, while the relative phase between the two solitons of molecule remains constant (intra-molecular phase), which is indeed the situation observed experimentally in Fig. 2. The trajectory of the SMC in the phase plane is shown in Fig. 5e: the circular path indicates the evolving relative phase at almost constant temporal separation.

As another interesting numerical observation, we show in Fig. 5b an oscillating-phase dynamics, obtained for *P* = 104 mW and *P*_sat_ = 7 W. The intra-molecular separation is 1.1 ps, and the inter-molecular separation is 3.9 ps, indicating possibly a stronger interaction between soliton-pair molecules than in the case of the sliding phase dynamics. Figure 5f depicts the evolutionary trajectories of the oscillating SMC in the interaction plane. A small-amplitude oscillation of the inter-molecular separation and the oscillation of the relative phases within the SMC can be noticed in Fig. 5f: this feature is also present in the experimental analysis, as shown by the slightly wobbling line of the first-order correlation in (experimental) Fig. 3d.

## Discussion

We interpret the results as revealing a major difference between the intra-molecular and inter-molecular bonds of the SMC. We first emphasise on the stronger intra-molecular bond between the two solitons that constitute each soliton-pair molecule. In the first SMC investigated, the strong intra-molecular bond corresponds to a dynamical attractor of focus type, whose strength also manifests in its low sensitivity to external perturbations. In contrast, the intermolecular bond, between the two soliton-pair molecules, operates over a distance thrice larger than the intra-molecular one and is characterised by a sliding relative phase, corresponding to an attractor of limit-cycle type. The latter constitutes a weaker attractor, which is more sensitive to environmental perturbations in general.

We remind that the elementary sliding-phase dynamics within a single soliton-pair molecule, was predicted in ref. ^23^ and confirmed experimentally, first through average measurements^24^ and then through real-time measurements^9,10^. We also point out that the experimental generation of multiple soliton pair molecules in a fibre laser cavity has been reported on several occasions. On the one hand, strongly bound soliton-pair molecules can behave as single pulsed-waveform entities. The overall pattern of soliton-pairs will depend on the interplay between the various interactions mechanisms that can take place in the laser cavity. For instance, if gain relaxation dominates, through the gain depletion and recovery mechanism^11^, a net repulsive force between the soliton-pair molecules can result in a stationary pattern of harmonic mode locking of soliton pairs^43,44^. In such a regular pattern, soliton pairs are equally distributed along the cavity. Nevertheless, the interaction based on gain depletion and recovery is weak and incoherent, resulting in an important pulse timing jitter. To overcome such large jitter, it is possible to design a laser cavity that incorporates a strong stabilisation mechanism, such as dissipative four-wave mixing^45^. On the other hand, when attractive forces dominate, two soliton pairs can interact strongly in a coherent way and form a stable and compact 4-soliton molecule^38^. In such a multi-soliton structure, the characterisation of the successive intra-molecular bonds of comparable strength is not a trivial issue in general.

The emphasis of the present article is on the coexistence of two different types of multi-soliton dynamics, acting upon intra-molecular and inter-molecular interactions, respectively, which give birth to a structure that can be compared to a molecular complex. We have found operational conditions in which a mode-locked fibre laser cavity generates two identical soliton-pair molecules, each consisting of a pair of solitons separated by a temporal separation of the order of the picosecond, and we have shown that the two soliton-pair molecules can interact in a coherent way at a significantly larger temporal separation. We have used a DFT-based spectral interferometry method to probe the interaction between the two soliton-pair molecules, showing for the first time a different dynamical nature of the intra-molecular and inter-molecular bonds. By properly adjusting the laser parameters, our laser setup generates diversified “allotropes” of SMCs, characterised by different internal dynamics. SMCs with sliding-phase and oscillating-phase dynamics have been characterised by the real-time spectral interferometry measurements, thus retrieving the dynamics of the major internal degrees of freedom of the complexes, namely the dynamics of the relative temporal and phase separations between the different soliton constituents. The analytical modelling and numerical simulations confirm the experimental observations and offer an additional insight into the understanding of the complex dynamics of SMCs.

By showing that soliton molecules can form various bonds according to the distance between soliton constituents, which we can manipulate, we consider that the present work opens the way to the manipulation of large-scale optical-soliton-molecule complexes and other compounds (macromolecules, crystals etc.), which were approached in past experiments without a developed real-time analysis. Reflecting the strong interest in the related area, let us mention an enthraling recent investigation within Kerr micro-resonators—a significantly different photonics platform, where soliton crystals featuring lattice defects have been found ^46^.

Based on the analogy between optical soliton molecules and chemical molecules, we can consider two major research avenues that we expect to attract a lot of attention in the near future.

One is to better understand and control the interactions among multiple optical solitons over larger temporal extensions, with the formation of larger molecular complexes, soliton macromolecules and crystals. As the internal degrees of freedom of the molecular complexes will be considerably increased (with, basically, two extra degrees of freedom per additional soliton), it will be interesting to see whether we can find complex dynamics that resemble the collective excitations of large chemical molecular structures. Within this direction of research, akin to the situation in supramolecular chemistry, long-range and short-range interactions will have to be introduced, which is likely to favour the build-up of structures having multiple scales^15,47,48^. We note recent developments showing how long-range interactions, for instance optomechanical^47^ or Casimir-like^48^ ones can be used to command the formation of large optical-soliton-molecule complexes.

Pattern formation with large number of solitons could trigger novel analogies with the structure of matter in general, maybe even beyond chemistry, due to the dissipative nature of soliton molecules.

For the moment, the large majority of investigated optical soliton molecules are linear ones, since they propagate in single-mode waveguides. The topic of ultrashort pulse generation and propagation in transverse multimode waveguides is currently driving considerable attention^49^. We anticipate that the study of spatiotemporal soliton molecules will develop shortly, noting a recent publication on this new topic^50^. With the enabling of the transverse spatial degrees of freedom, the topic of optical soliton molecules will represent an even closer analogy with the molecules of chemistry, with three-dimensional structures studied from the structural conformation point of view, as well as from the dynamical point of view, which is a topic of high stakes in chemistry (protein dynamics for instance).

Finally, by combining the two previous directions, namely combining short and long-range interaction (strong and weak binding), as well as more spatiotemporal dimensions of the soliton propagation, we will have the possibility to assemble the equivalent of three-dimensional supramolecular structures—which includes DNA and viruses in the chemical world. Naturally, the challenging real-time characterisation of ultrafast spatiotemporal photonic structures will pose specific issues, so that considerable technical advances are expected in this area.

## Methods

### Experimental setup

We investigate the dynamics of stable dissipative patterns made of four interacting dissipative solitons that are generated from an erbium-doped fibre (EDF) ring laser, mode-locked by the nonlinear polarisation evolution (NPE) technique^39,40^. Within NPE, the nonlinear transfer function is tuned by adjusting the orientation of the intracavity wave plates displayed in Fig. 1 and can thus act as a quasi-instantaneous saturable absorber. The fibre laser configuration is almost the same as in ref. ^9^, except for the length of single-mode fibre (SMF). The experimental setup is sketched in Fig. 1. The total length of SMF in our experiments is 3.4(4.8) m for the 2 + 2 soliton molecular complex with sliding phase (oscillating phase, respectively). Employing a 0.55-m EDF, the laser yields a net anomalous dispersion *β*_2_ = −0.33(−0.52) ps^2^ at the 1.55-μm wavelength. Self-starting mode locking with multi-pulses occurs at around 400 mW of pump power, but mode locking can be maintained at reduced pump powers down to a threshold of 165 mW. The fundamental repetition frequency of the cavity is 47.94 (35.77) MHz, corresponding to a roundtrip time of 20.9 (27.96) ns. The roundtrip time constitutes a window within which the pulses are stretched in the frame of the DFT measurement technique^30^. The latter is implemented by propagating pulses through a 6345-m long dispersion-compensation fibre (DCF). The DCF has a normal dispersion of −108 ps.nm^−1^.km^−1^ at 1.55 μm, so as to provide a total accumulated dispersion of 769 ps^2^. The signal is detected with a high-speed 45-GHz photodiode plugged into a 6-GHz 40-GSa/s real-time oscilloscope. Thus, the scale of wavelength-to-time mapping is 1.46 nm per ns, and the electronic-based spectrum resolution of our system is 0.3 nm.

Let us comment on the experimental procedure which allows to generate several types of SMCs in a reproducible way. The pump power is the main parameter controlling the number of pulses, whereas the intracavity wave plates allow to fine tune the interactions among pulses. As stated in the Results section, the nonlinear dynamics of ultrashort pulse features important hysteresis and multi-stability. Therefore, the suitable control parameters are not unique and not independent, they are found in a relatively wide range. For instance, the 2 + 2 oscillating-phase SMCs are found in a 210–280 mW pump power range, also depending on the wave plate settings. To generate a given type of SMC, we move the pump power from higher values (350–400 mW) down to the appropriate range, and tune slowly the wave plates, monitoring the evolution of the multi-pulse structures using real-time, as well as average spectral and temporal measurements. Multiple solitons have a major tendency to form soliton pairs, with a relatively strong bond. Therefore, multiple soliton pairs usually appear first. Then, they can be manipulated as units, through a fine tuning of the control parameters that affect their long-range interactions. This way, a given type of 2 + 2 SMC can be generated, for instance an oscillating-phase SMC. Such 2 + 2 SMC is not self-starting: if we switch off, then switch on the laser, the SMC will generally not appear. To repeat the experiment, we have to follow the whole hysteretic procedure with pump power and waveplate orientation as control parameters. The experiment is reproducible in the sense that a given type of SMC can be found repeatedly over days and months. However, the fine structure of the complexes, such as the specific pulse separations, can change from one experimental run to the next. This is due to the large amount of multi-stability, a general feature associated with the existence of a fine structure of multi-soliton attractors. Such fine structure was pointed out in early soliton-pair experiments, and confirmed numerically^51^. The fine structure of SMCs, involving more than two soliton pulses, is considerably more complex. To illustrate this feature, we provide other examples of oscillating-phase SMCs in the Supplementary Information (See Suppl. Figs. 2 and 3 and Suppl. Note 2).

### Phase retrieval

We illustrate the internal phase retrieval in the case of a single soliton-pair molecule. The principle can be extended to molecules containing a larger number of solitons, under some conditions. We assume that the soliton-pair molecule consists of two pulses of identical shape and amplitude, which makes their relative temporal separation *τ* and phase *φ* to be considered as the two internal degrees of freedom. The soliton-pair molecule electric field envelope reads:

1 $$E_{{\mathrm{sp}}}\left( t \right) = E_0\left( {t - \frac{\tau }{2}} \right) + E_0\left( {t + \frac{\tau }{2}} \right) \cdot e^{ - i\varphi }$$

where *E*_0_ is the single soliton electric field profile. In the frequency domain, the soliton pair yields the following spectral intensity:

,2 $$I(\omega ) = 2I_0(\omega )[1 + \cos \left( {\omega \tau - \varphi } \right)]$$

where *I*_0_(*ω*) = |*E*_0_(*ω*)|^2^ represents the optical spectrum of a single soliton. The pulse separation *τ* determines the fringe period of the modulated spectral intensity and the relative phase can be retrieved as *φ* = 2*π δν*/*∆ν*, where *δν* is the frequency offset between the central frequency of the carrier-envelope and the frequency at the maximal spectral intensity. However, this method requires a precise determination of the central frequency. In addition, with more than two pulses, the interference spectrum becomes more complicated, making access to an accurate *δν* more difficult. By Fourier-transforming the DFT single-shot spectrum, we obtain a first-order autocorrelation function, known as the temporal coherence function, which can be expressed as:

3 $${\mathrm{\Gamma }}\left( {\tau \prime } \right) = 2{\int} {I_0\left( \omega \right)e^{i\omega \tau \prime }\mathrm{d}\omega } + e^{-i\varphi }{\int} {I_0\left( \omega \right)e^{i\omega (\tau + \tau \prime )}\mathrm{d}\omega } + e^{ i\varphi }{\int} {I_0\left( \omega \right)e^{i\omega \left( {\tau - \tau \prime } \right)}\mathrm{d}\omega }$$

We define the three contributions in Eq. (3) through ${\mathrm{\Gamma }}\left( {\tau \prime } \right) \equiv G_{{\mathrm{cent}}}\left( {\tau \prime } \right) + G_{{\mathrm{left}}}\left( {\tau \prime } \right) + G_{{\mathrm{right}}}\left( {\tau \prime } \right)$ where the central part term $G_{{\mathrm{cent}}}\left( {\tau \prime } \right)$ represents the incoherent superposition of the optical intensity of the pulses and the terms $G_{{\mathrm{left}}}\left( {\tau \prime = - \tau } \right)$ and $G_{{\mathrm{right}}}\left( {\tau \prime = \tau } \right)$ contain the phase information. We rewrite $G_{{\mathrm{right}}}\left( {\tau \prime = \tau } \right) \equiv P_0e^{ i\varphi }$ and extract the phase *φ* from the imaginary part of log_e_[*G*_right_(*τ’* *=* *τ*)]. We symmetrise the phase retrieval procedure by using both $G_{{\mathrm{left}}}\left( {\tau \prime = - \tau } \right)$ and $G_{{\mathrm{right}}}\left( {\tau \prime = \tau } \right)$.

The interference spectral intensity pattern is dependent on the phase difference, with a period of 2*π*, therefore the retrieved phase can be added 2*kπ* by continuity, a procedure called phase unwrapping. All the retrieved relative phases of this work are obtained through this procedure. Note also that to unwrap the phase correctly along thousands of successive cavity roundtrips, one needs to take precisely into account the cavity roundtrip time. This point is illustrated in Supplementary Method 1. The precision of the retrieval is not affected significantly by small sidelobe artefacts related to the fast-electronic acquisition, see the Suppl. Note 3.

### Numerical simulations

In the lumped propagation model, each component of the cavity is modelled by a separate equation, and the pulse propagation follows a concatenated sequence representing the different cavity elements. The pulse propagation in the optical fibres is modelled by a generalised nonlinear Schrödinger equation, in the scalar approach, which takes the following form ^52^:

4 $$\frac{{\partial {\it{\psi }}}}{{\partial z}} = - \frac{{\it{\alpha }}}{2}{\it{\psi }} - i\frac{{{\it{\beta }}_2}}{2}\frac{{\partial ^2{\it{\psi }}}}{{\partial ^2t}} + g\left( z \right){\it{\psi }} + i{\it{\gamma }}\left| {\it{\psi }} \right|^2{\it{\psi }}$$

where *ψ* is the slowly varying electric field moving at the group velocity along the propagation coordinate *z*, and *α, γ, β*_2_ are the linear loss, Kerr nonlinearity and second-order dispersion coefficients, respectively. We used the measured dispersion values for *β*_2_ and the calculated nonlinear coefficients *γ* = 3.6 × 10^−3 ^W^−1 ^m^−1^ and 1.3 × 10^−3 ^W^−1 ^m^−1^ for EDF and SMF, respectively.

In the SMF, we set *g* = 0, while in the EDF, the gain function *g*(*z*) is obtained by using a two-effective-level amplifier rate equation model. The EDF is doped with *N*_0_ erbium ions per unit volume. Both the pump and the signal co-propagate in the LP_01_ fundamental transverse mode of the EDF. The power distribution along the fibre is then given by the following rate equations ^53–56^:

5a $$\frac{{\mathrm{d}P_{\mathrm{p}}}}{{\mathrm{d}z}} = - \sigma _{\mathrm{p}}^{\mathrm{a}}n_1\left( z \right)N_0\Gamma _{\mathrm{p}}P_{\mathrm{p}}(z)$$

5b $$\frac{{\mathrm{d}P_{\mathrm{s}}}}{{\mathrm{d}z}} = \left[ {\sigma _{\mathrm{s}}^{\mathrm{e}}\left( {\nu _{\mathrm{s}}} \right)n_2\left( z \right) - \sigma _{\mathrm{s}}^{\mathrm{a}}\left( {\nu _{\mathrm{s}}} \right)n_1\left( z \right)} \right]N_0\Gamma _{\mathrm{s}}\left( {\nu _{\mathrm{s}}} \right)P_{\mathrm{s}}(z)$$

where *P*_p_ and *P*_s_ designate the pump and signal power at a position *z* in the fibre, $\sigma _{\mathrm{p}}^{\mathrm{a}}$ is the absorption cross section of erbium ions at the 980-nm pump wavelength, $\sigma _{\mathrm{s}}^{\mathrm{a}}$ and $\sigma _{\mathrm{s}}^{\mathrm{e}}$ are the absorption and emission cross sections at the signal optical frequency *v*_s_, *n*_1,2_(*z*) represent the fractional erbium population distribution between ground and excited states, and Γ_s,p_ are the modal overlap factors. We take $\sigma _{\mathrm{p}}^{\mathrm{a}} = 2.17 \times 10^{ - 25}{\mathrm{m}}^2$, the cross-section frequency dependence of $\sigma _{\mathrm{s}}^{\mathrm{a}}$ and $\sigma _{\mathrm{s}}^{\mathrm{e}}$ are taken from^57^, and *N*_0_ = 6.8 × 10^24^ m^−3^. After calculating the steady-state values of the population densities, we solve equations (5a) and (5b) by means of the standard Runge-Kutta algorithm and obtain the gain coefficient amplitude as^58^: $g\left( {z,P_{{\mathrm{av}}},\nu _{\mathrm{s}}} \right) = \mathrm{d}({\mathrm{ln}}P_{\mathrm{s}})/\mathrm{d}z$, where $P_{{\mathrm{av}}}\left( z \right) = \frac{1}{{\tau _{{\mathrm{RT}}}}}\mathop {\int }\limits_0^{\tau _{{\mathrm{RT}}}} \left| {\psi (t,z)} \right|^2\mathrm{d}t$. Therefore, the calculated gain coefficient includes the saturation effect, as well as the spectral and longitudinal dependences of the amplification process in the EDF.

The effective nonlinear saturation involved in the NPE mode locking technique is modelled by the following instantaneous transfer function: *P*_*o*_ = *T* × *P*_*i*_ where *T* ≡ *T*_0_ + *∆T*×*Pi* /( *P*_*i*_ + *P*_sat_), describes the transmission of the saturable absorber, *P*_*i*_ (*P*_o_) being the instantaneous input (output) optical power, normalised as $P\left( {z,t} \right) = \left| {\psi (z,t)} \right|^2$. As typical values, we take *T*_0_ = 0.70 for the transmissivity at low signal and *∆T* = 0.30 as the absorption contrast. We emulate the experimental situation by manoeuvring the control parameters of the cavity. Particularly, we tweak the mode-locking conditions by tuning the pump power and the saturation power *P*_sat_. The phase is estimated using standard phase retrieval algorithm, which takes into account the phase jump by properly unwrapping the retrieved phase.

### Code availability

The simulation code, which was originally developed in the frame of refs. ^9^ and ^53^, is available upon reasonable written request, which excludes any commercial interest.

## Supplementary information

Supplementary Information

Description of Additional Supplementary Files

Supplementary Movie 1

Supplementary Movie 2

## Data Availability

The data supporting the plots within this paper and other findings of this study are available from the corresponding author upon reasonable request.
