# Supplementary material for: Optical soliton molecular complexes in a passively mode-locked fibre laser
Source: Nat Commun. 2019 Feb 19;10:830. doi: 10.1038/s41467-019-08755-4 (PMC6381207; doi:10.1038/s41467-019-08755-4)
Supplement: Supplementary file 1 — Supplementary Information [file 41467_2019_8755_MOESM1_ESM.pdf]

Supplementary information

Optical soliton molecular complexes in a passively mode-locked fibre laser

Z. Q. Wang, K. Nithyanandan, A. Coillet, P. Tchofo-Dinda & Ph. Grelu

Supplementary Note 1 | Animated graphical representation of the soliton molecular complex.

The soliton molecular complex (SMC) is an ultrashort optical structure that binds two soliton-pair optical molecules. Each soliton-pair molecule consists of a bound state made of two single soliton pulses that have an identical intensity profile. The internal degrees of freedom of this SMC are the relative temporal separations τ_i and phases φ_i between successive solitons. To better understand the internal dynamics of SMCs, the solitons are represented by phasors (See Supplementary Figure 1a), distributed along a temporal axis. In our convention, the leading soliton is a fixed phasor, pointing upward in the diagram, which serves as a reference for the succeeding phasors. Note that this representation does not stand for the polarization of the pulses, as this degree of freedom is frozen in our laser experimental setup, by virtue of the intracavity polarizer. Supplementary Figure 1b and Supplementary Figure 1c link to graphical videos illustrating the two classes of dynamics discussed in the article, namely, sliding-phase and oscillating-phase SMCs, respectively.

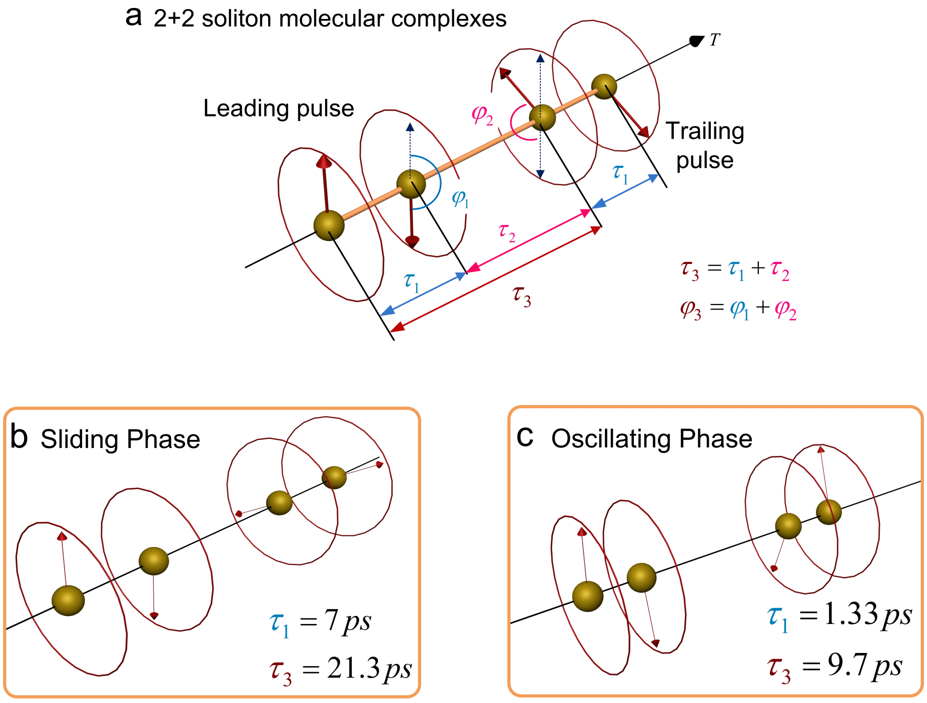

Supplementary Figure 1 | Graphical representation of the dynamics of the internal degrees of freedom of a soliton molecular complex. **a** Definition of the internal degrees of freedom, namely the relative temporal separations τ_i and phases φ_i in a phasor representation. **b** Sliding phase dynamics. See the corresponding Supplementary Movie

1. **c** Oscillating-phase dynamics. See the corresponding Supplementary Movie 2. The values of τ_1 and τ_3 are indicated in **b** and **c**, in correspondence to the experimental soliton molecular complexes analysed in the article.

Supplementary Note 2 | On the possible influence of a quasi-continuous-wave spectral component on the dynamics of soliton molecular complexes.

The presence of a quasi-continuous wave (CW) radiation on the soliton molecular complex (SMC) spectrum is found to have a minor impact on the existence of the quasi-stationary oscillating-phase dynamics that we can prepare and observe. To illustrate this assertion, we present, on Supplementary Figure 2 and Supplementary Figure 3, two additional experimental cases of SMC with, and respectively without, a CW radiation peak on the spectrum. In Supplementary Figure 2a, the CW peak is apparent on the average optical spectrum, recorded by a conventional spectrum analyser, but does not appear in the single-shot spectral recording, since it is widely distributed in the time domain. The recording of these two additional experimental cases indicates that such CW component has a negligible impact on the existence of intra- and inter- molecule phase dynamics in the oscillating case. In both cases, it is found that, whereas the phase oscillation between solitons within each soliton molecule is a regular one, some long-term fluctuations appear for the phase oscillation between the two soliton pairs that constitute the molecular complex. We attribute this effect to the weaker bond nature, which is therefore more sensitive to environmental perturbations, between the multiple soliton pairs that make up the complex.

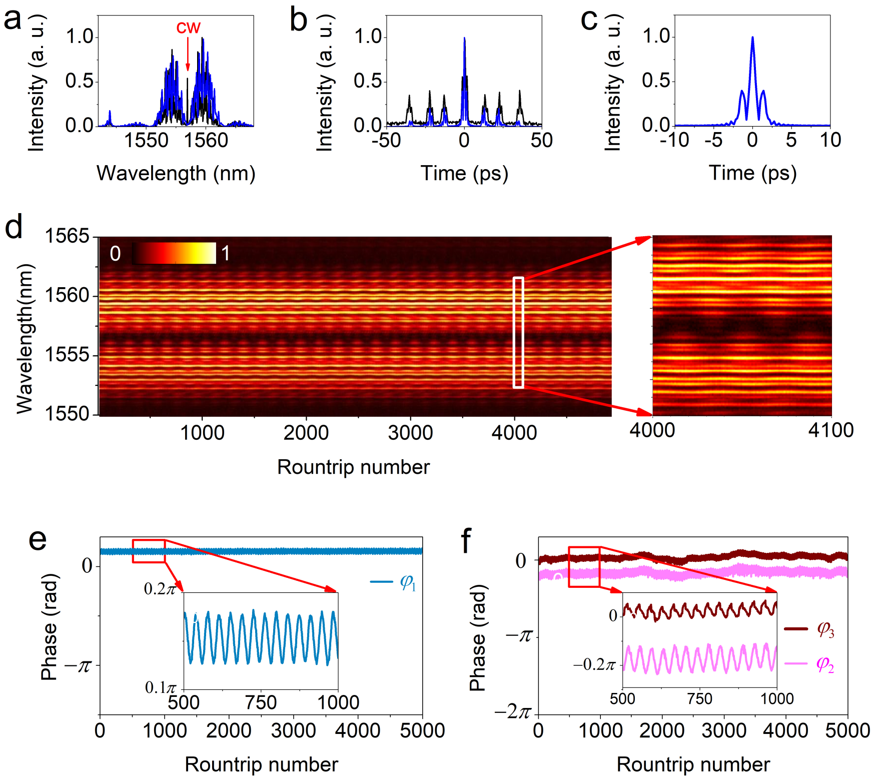

Supplementary Figure 2 | Soliton molecular complex with oscillating-phase dynamics in presence of a quasi-continuous-wave additional radiation. **a** Average (in black) and single-shot (in blue) optical spectrum. **b** First-order (in blue) and second-order (in black) optical autocorrelation. **c** Zoom-in showing the central correlation peaks of the first-order autocorrelation. **d** Evolution of single-shot optical spectra recorded after each roundtrip, over 5000 roundtrips (spectral intensity in colour scale), with a magnification between roundtrips 4000 and 4100. **e**

Retrieval of the relative phase φ_1 and its oscillating dynamics. **f** Retrieval of the oscillating relative phases φ_2 and φ_3.

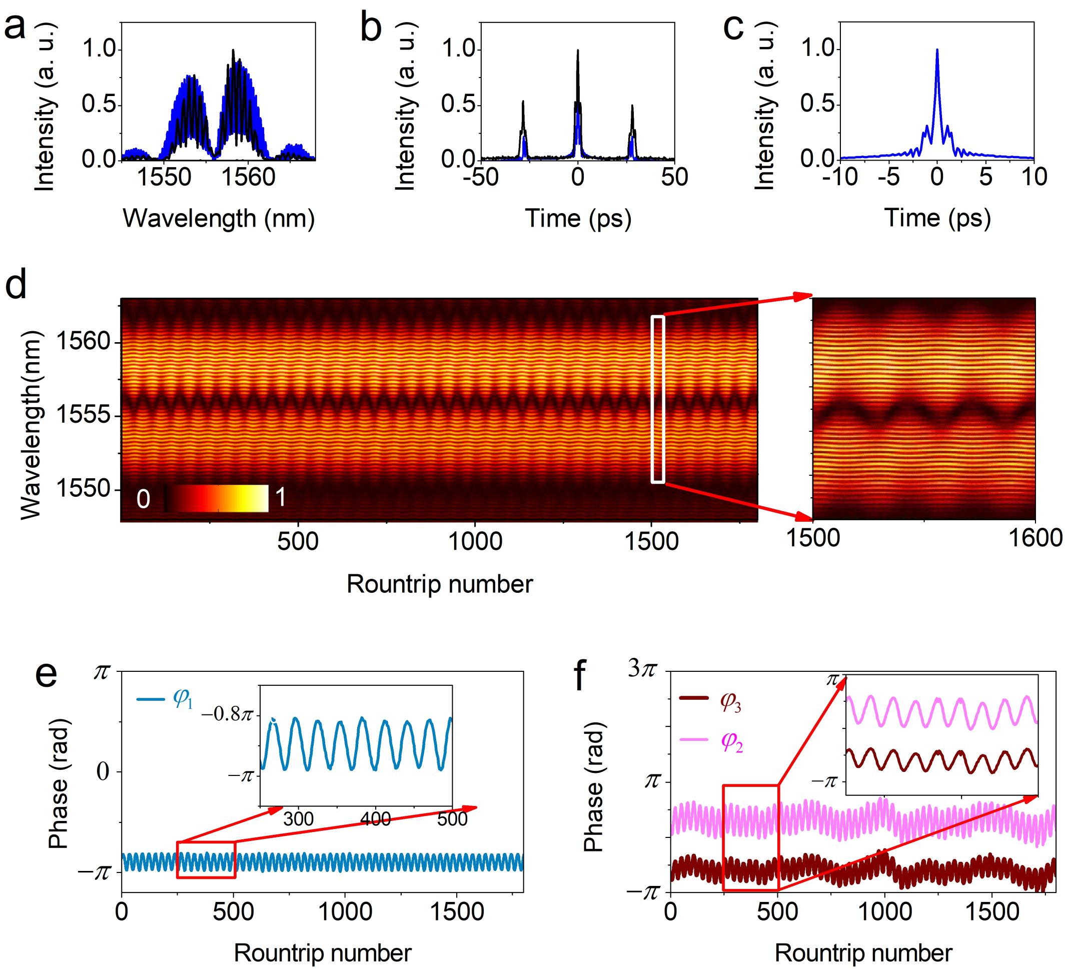

Supplementary Figure 3| Soliton molecular complex with oscillating-phase dynamics without quasi-continuous-wave radiation. **a** Average (in black) and single-shot (in blue) optical spectrum. **b** First-order (in blue) and second-order (in black) optical autocorrelation. **c** Zoom-in showing the central correlation peaks of the first-order autocorrelation. **d** Evolution of single-shot optical spectra recorded after each roundtrip, over 1800 roundtrips (spectral intensity in colour scale), with a magnification between roundtrips 1500 and 1600. **e** Retrieval of the relative phase φ_1 and its oscillating dynamics. **f** Retrieval of the oscillating relative phases φ_2 and φ_3.

Supplementary Method | Importance of the calibration of roundtrip times during the phase retrieving process.

A slight inaccuracy of the roundtrip time measurement during the analysis of the dispersive Fourier-transform recording causes an apparent movement of the centre of mass of the spectrum. Once the fast Fourier-transform operation is performed, this movement is translated into a phase drift, round-trip after round-trip, that is proportional to the time-delay of the retrieved first-order auto-correlation trace. Even with a misalignment in the range of a few femtoseconds, which is less than a one-in-a-million difference, an artificial phase drift will appear after several hundreds of cavity roundtrips. To illustrate this parasitic effect in the retrieval, the effect of a 10-

femtoseconds step change in the roundtrip time is shown in the Supplementary Figure 4, clearly impacting the retrieved relative phases φ_1, φ_2 and φ_3 within the soliton molecular complex. These phases also correspond to three different delays between solitons, therefore producing different phase slopes. To conclude on this point, considering a follow up of the dynamics over thousands of cavity roundtrips, the roundtrip time should be accurate to the level of ten femtoseconds or below to make sure that no artificial phase drifts appear.

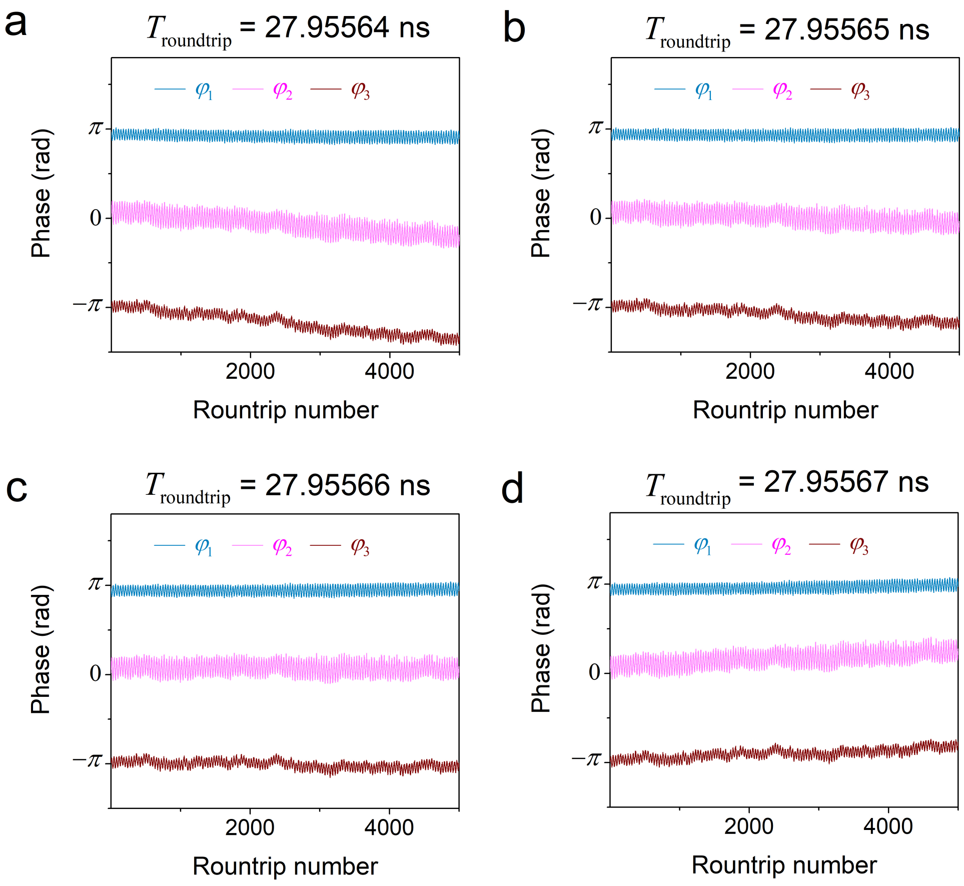

Supplementary Figure 4| Effect of the roundtrip time precision on the retrieval of the internal phases. The cavity roundtrip time is fixed in the experiment. Any slight mismatch in assessing the value of the cavity roundtrip time T_{rountrip}, which is used to process a given recording made of successive single-shot spectra, will lead to artificial drifts in the evolution of the relative internal phases φ_1, φ_2 and φ_3. For illustration, the increment between **a, b**, **c** and **d** is 10 fs.

Supplementary Note 3. Small sidelobe artefacts on the retrieved first-order autocorrelation trace.

The first-order optical autocorrelation traces displayed on Fig. 3 of the main article reveal the presence of sidelobes on each sides of the main peaks. To find out the origin of such small peak artefacts, which are not observed on the averaged second-order optical autocorrelation traces, we calculated the first-order autocorrelation traces from the Fourier transform of single-shot optical spectra under different conditions. We found that the additional peak artefact originates from a slight imperfection of our fast-electronics detection scheme. Namely, we noticed the existence of a small imperfect impedance matching, which combines a limited vertical dynamic range with the relatively high noise of fast electronic detectors and acquisition systems. These are recurrent issues in fast electronic

measurements. They can be improved in principle, but hardly eliminated in the case of weak signals. This issue represents one of the actual limitations of the time-stretch dispersive Fourier-transform (DFT) technique. In the Supplementary Figure 5, to show that this is a recurrent artefact, we record and analyse single-shot optical spectra for the case of a stable single soliton traveling around the laser cavity. The multi-shot (averaged) autocorrelation trace shows no sign of sidelobes or pedestal features (see the inset of the Suppl. Fig. 5), attesting a clean single pulse regime. In contrast, the first-order autocorrelation trace exhibits a symmetrical pair of low-amplitude bounces near the central peak. We anticipate that the coming new generation of fast oscilloscopes, by increasing significantly the signal to noise ratio for weak amplitude signals and the quality of impedance matching adjustment, will improve the performance of DFT-based measurements, which will be required to analyse larger soliton molecular complexes. Nevertheless, we emphasize on the fact that these small peak artefacts did not have any significant impact on the analysis of the soliton molecular complexes discussed in the main article. We also checked the internal consistency of the phase retrieval, which $\varphi_3 = \varphi_1 + \varphi_2 \pm 2m\pi$, where m is an integer.

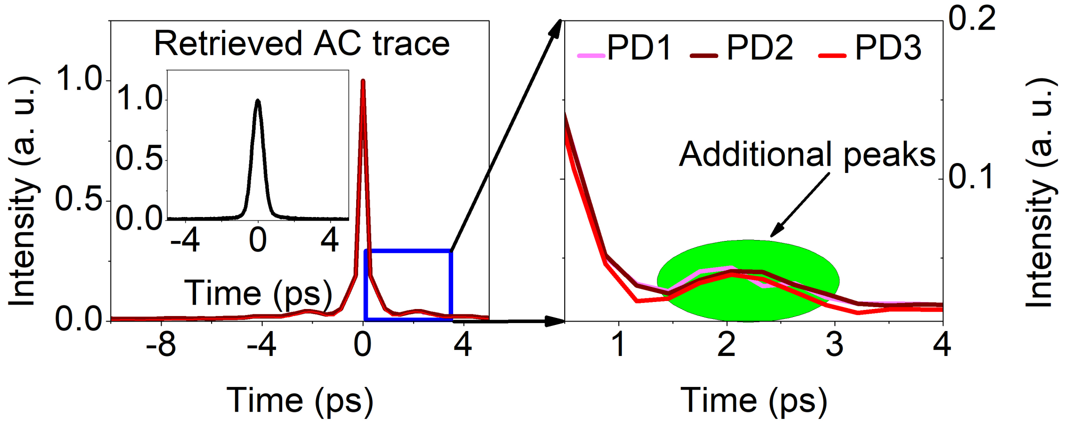

Supplementary Figure 5| Peak artefacts in the first-order autocorrelation traces. Example of retrieved first-order autocorrelation (AC) trace, calculated from a recorded single-shot spectrum, when the laser is mode locked with a single pulse state. Inset: multi-shot-averaged second-order AC trace. Zoom-in of the peak artefact, whose amplitude is typically around 4% of the main peak. Three different photodiodes (PD) were used, with different bandwidths: 12GHz (PD1), 12.5GHz (PD2) and 45GHz (PD3).
