# Supplementary material for: Optical soliton molecular complexes in a passively mode-locked fibre laser
Source: Nat Commun. 2019 Feb 19;10:830. doi: 10.1038/s41467-019-08755-4 (PMC6381207; doi:10.1038/s41467-019-08755-4)
Supplement: Supplementary file 2 — Description of Additional Supplementary Files [file 41467_2019_8755_MOESM2_ESM.pdf]

Description of Additional Supplementary Files

File Name: Supplementary Movie 1
Description: Video representation of the sliding-phase dynamics of a 2+2 soliton molecular complex using the phasor representation

File Name: Supplementary Movie 2
Description: Video representation of the oscillating-phase dynamics of a 2+2 soliton molecular complex using the phasor representation
